# Electrochemical camera chip for simultaneous imaging of multiple metabolites in biofilms

**DOI:** 10.1038/ncomms10535

**Published:** 2016-01-27

**Authors:** Daniel L. Bellin, Hassan Sakhtah, Yihan Zhang, Alexa Price-Whelan, Lars E. P. Dietrich, Kenneth L. Shepard

**Affiliations:** 1 Department of Electrical Engineering, Columbia University, 1300 S.W. Mudd Building, 500 West 120th Street, New York, New York 10027, USA; 2 Department of Biological Sciences, Columbia University, Fairchild Center, 1212 Amsterdam Avenue, New York, New York 10027, USA

## Abstract

Monitoring spatial distribution of metabolites in multicellular structures can enhance understanding of the biochemical processes and regulation involved in cellular community development. Here we report on an electrochemical camera chip capable of simultaneous spatial imaging of multiple redox-active phenazine metabolites produced by *Pseudomonas aeruginosa* PA14 colony biofilms. The chip features an 8 mm × 8 mm array of 1,824 electrodes multiplexed to 38 parallel output channels. Using this chip, we demonstrate potential-sweep-based electrochemical imaging of whole-biofilms at measurement rates in excess of 0.2 s per electrode. Analysis of mutants with various capacities for phenazine production reveals distribution of phenazine-1-carboxylic acid (PCA) throughout the colony, with 5-methylphenazine-1-carboxylic acid (5-MCA) and pyocyanin (PYO) localized to the colony edge. Anaerobic growth on nitrate confirms the O_2_-dependence of PYO production and indicates an effect of O_2_ availability on 5-MCA synthesis. This integrated-circuit-based technique promises wide applicability in detecting redox-active species from diverse biological samples.

## Abstract (web summary)

Much remains to be understood about the biochemical basis of microbial community behaviour in biofilms. Here the authors describe a novel electrochemical camera chip that allows simultaneous spatial imaging of multiple metabolites that are crucial to community behaviour within whole microbial biofilms.

## Graphical abstract

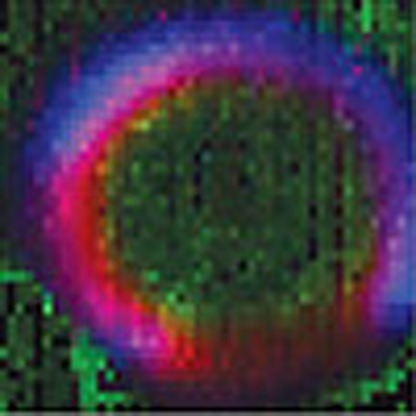

Redox transformations of metabolites are fundamental to life and can act as signatures for distinct physiological processes and organisms. The functions of discrete multicellular structures are tied to metabolic processes that control their formation^1^,^2^. Monitoring the spatiotemporal distribution of metabolites as they are produced by cellular communities during development can not only reveal the biochemical processes involved but also provide clues as to the mechanisms by which cells regulate these processes. Unfortunately, efficient methodologies for metabolite sensing, particularly using non-invasive methods, are severely lacking.

We investigate a platform to image phenazines, a class of redox-active metabolites produced by the gram-negative opportunistic pathogen *Pseudomonas aeruginosa* PA14. Phenazines, which strongly affect development of *P. aeruginosa* PA14 biofilms^3^,^4^,^5^, have a core heterocyclic structure that is modified with functional groups, giving rise to a diversity of phenazine variants with different chemical properties, including redox potential^6^,^7^. These compounds are produced when cells are present at high density^6^ and can act as intercellular signals^8^ and as alternate electron acceptors to balance the intracellular redox state^9^,^10^. Wild-type *P. aeruginosa* colony biofilms produce at least four phenazines, including phenazine-1-carboxylic acid (PCA), 5-methylphenazine-1-carboxylic acid (5-MCA), pyocyanin (PYO) and phenazine-1-carboxamide (PCN)^11^,^12^,^13^ (Fig. 1), which engage in two-electron redox reactions. The complement, redox states and distribution of phenazines in a *P. aeruginosa* colony are likely affected by many factors including the expression of biosynthetic enzymes, the availability of substrates for biosynthesis and the environmental reduction potential.

Available techniques for directly imaging phenazines as a representative family of metabolites include two-photon excitation microscopy, imaging mass spectrometry (IMS) and scanning electrochemical microscopy (SECM). Two-photon excitation microscopy has the advantage of non-invasiveness and the potential for large-area, high-frame rate optical imaging but suffers from poor sensitivity and specificity of detection^14^, which has prevented imaging of biofilms in practice. Phenazines have been detected in biofilms using IMS (refs 15, 16, 17). A drawback of IMS in these studies is the extensive sample treatment required. Though recent advances in IMS may alleviate sample pretreatment requirements^18^, mass spectrometry approaches still necessitate bulky and expensive instrumentation, and quantifying the chemical species sensed is currently not possible with most IMS techniques^19^.

Electrochemical techniques are an attractive choice for detecting phenazines due to their strong redox activity, and the use of this electrochemical activity for detection and quantification could be applied to many metabolites in addition to phenazines. Electrochemical detection of phenazines has been demonstrated in liquid cultures of *P. aeruginosa*^20^,^21^,^22^,^23^,^24^,^25^ and in biofilms^26^, though the latter study did not attempt detection with spatial resolution. SECM has been used for spatially resolved detection of phenazines in *P. aeruginosa* biofilms^27^ and aggregates^28^. SECM employs a scanning working electrode to measure electrochemical currents as a function of position and has an advantage over other methods in that it enables three-dimensional profiling of individual species. However, due to the constant potential at the working electrode, SECM is not able to simultaneously detect species with different redox potentials in practice, in contrast to potential sweep methods. SECM cannot be effectively combined with potential sweep methods because the length of a single potential sweep experiment (on the order of seconds) and the presence of only one measurement probe would require prohibitively long analysis times for imaging applications.

We have previously reported on an integrated circuit (IC) capable of spatially resolved electrochemical detection of multiple phenazines from *P. aeruginosa* colony biofilms^13^. Array size for this design was only 60 electrodes within a 3.25 mm × 0.9 mm area, preventing formation of full two-dimensional images of entire colonies. Imaging of colonies was done on an agar layer, horizontal diffusion through which significantly impacted spatial resolution. Furthermore, frame rates were only 8 s per electrode. Here we present a new IC with a dramatically larger working electrode array and larger number of parallel output channels, enabling whole-colony biofilms to be electrochemically imaged for the first time. This IC can be produced at a manufacturing cost of <$5 and is controlled by a graphical user interface requiring little technical expertise to operate. We use this electrochemical camera chip combined with square-wave voltammetry (SWV), a potential sweep method, to simultaneously image multiple phenazines in two dimensions in *P. aeruginosa* PA14 colony biofilms and investigate the effects of O_2_ availability on phenazine production, distribution and chemistry. Here, colonies are supported by agar-soaked membranes <15-μm thick placed directly on top of the chip. The reduced distance between the colonies and the electrodes ensures that phenazines do not significantly diffuse laterally before detection at the electrodes, thus achieving a spatial resolution of 225 μm, limited only by electrode pitch (Supplementary Fig. 1). Frame rates in excess of 0.2 s per electrode are supported, more than adequate for the temporal dynamics studied.

## Results

### Electrochemical camera chip

Key to this work is the development of an electrochemical camera chip, a custom-designed IC fabricated in a 0.25-μm complementary metal-oxide-semiconductor (CMOS) process (Fig. 2a,b). As such, this work demonstrates the unique capabilities that CMOS technology provides in allowing for dense arrays of electrodes for electrochemical analysis of biological systems in which close proximity can be achieved between the biological specimen and the IC. The 1-cm-by-1-cm chip features an 8-mm-by-8-mm array of 1,824 gold integrated electrodes, each 100 μm × 100 μm, in a 48 × 38 grid. Working electrodes are multiplexed to 38 parallel output channels each featuring a programmable transimpedance amplifier (Supplementary Figs 2–4 provide further circuit design details). The chip also features an integrated control amplifier driving a potentiostat circuit, consisting of the working electrode array, an integrated gold counter electrode, and an integrated silver/silver-chloride quasireference electrode. Here, *P. aeruginosa* PA14 colonies are grown on thick agar plates, with an interposing track-etched membrane allowing the colony to be transferred directly onto the chip (Fig. 2c). Growth on this membrane does not significantly affect colony morphology.

### Detection of phenazines

We have previously shown that with SWV, it is possible to distinguish PCA, PYO and 5-MCA on secretion by a *P. aeruginosa* PA14 colony into agar and detect their distribution over a cross-section of the colony base^13^. Colony growth is initiated by pipetting a 5-μl droplet of a cell suspension onto a membrane placed on agar-solidified medium. After approximately 2 days of growth, the membrane and colony are transferred to the electrochemical camera chip for analysis. We previously found that in wild-type biofilms, PYO levels peaked at a location near the edge of the colony^13^. In addition, in a phenazine-overproducing mutant, we found spatial modulation in the proportions of two phenazines produced by the colony.

Using the electrochemical camera chip, we are able to simultaneously image for the first time the spatial distribution of three different phenazines in whole-colonies *in situ.* Using both reductive and oxidative SWV (where ‘reductive' and ‘oxidative' refer to the direction of the applied voltage signal) enables us to ascertain the phenazine redox state, a major advantage of the IC-based platform over other imaging modalities such as IMS. We find that peak current values on square-wave voltammograms are linearly related to concentration^13^ and can be calibrated, allowing images to be presented in terms of phenazine concentration. We, furthermore, show that the baseline square-wave voltammogram from the Δ*phz* mutant shows no peaks or other features (Supplementary Fig. 5). Calibration curves (Supplementary Figs 6–8) are determined by averaging among 120 electrodes. Variability between currents measured at different electrodes for a given concentration generally increases with increasing concentration.

To demonstrate the whole-colony imaging capabilities of our IC-based imager, we first analyse a series of biofilms formed by *P. aeruginosa* PA14 mutants producing different combinations of phenazines. We focus on the production and distribution of 5-MCA and PYO, two phenazines that are uniquely produced by the pathogen *P. aeruginosa* among the dozens of known phenazine-producing bacterial species^29^. Characterization of *P. aeruginosa* 5-MCA production by conventional methods has been historically difficult due to its reactivity, while PYO is considered the phenazine of highest pathological significance due to its production during *P. aeruginosa* host colonization. For simplicity, all strains used in this study were generated in the Δ*phzH* background and lack the ability to produce PCN. (Production of PCN does not significantly affect the 5-MCA and PYO production patterns discussed in the remainder of this study (Supplementary Figs 9 and 10)). We start with Δ*phzHM*, for which PCA is the only expected phenazine product^11^. Figure 3a shows reductive SWV-based images of a Δ*phzHM* biofilm grown for 2 days. Reductive SWV reveals two compounds, one with a peak current at −500 mV, which corresponds to that observed for pure PCA in solution (Supplementary Fig. 11) and one with a peak current at approximately −300 mV (Fig. 3b,c). The latter peak is absent from reductive SWV traces generated for the Δ*phz* biofilm (Supplementary Fig. 5). SWV of pure phenazine-1,6-dicarboxylic acid, an alternate product of the PCA-generating enzyme PhzG, confirms that the −300-mV peak in Δ*phzHM*-colony SWV traces cannot be attributed to this compound (Supplementary Fig. 12)^30^. We, therefore, believe that it represents another product of the PhzA-G-dependent portion of the phenazine biosynthetic pathway. In the Δ*phzHM* biofilm, PCA is produced both at the centre and the edges of the colony. A darker zone, indicating lower concentrations of PCA, is visible between the edge and the centre (Fig. 3a). The zone of lower PCA concentrations seems to overlap with a zone of higher concentrations of the unidentified compound, suggesting an inverse relationship in production of the two. The peak generated by the unidentified compound overlaps with the PCA peak in oxidative SWV traces at −300 mV (Supplementary Fig. 13), making it indistinguishable there. Figure 3a demonstrates some asymmetry in the distribution of phenazines that localize to the colony periphery that is characteristic in many of the images. We suspect that this arises from unevenness in the agar that distributes cells unevenly in the 5-μl cell suspension droplet used as inoculum.

**Figure 1:**
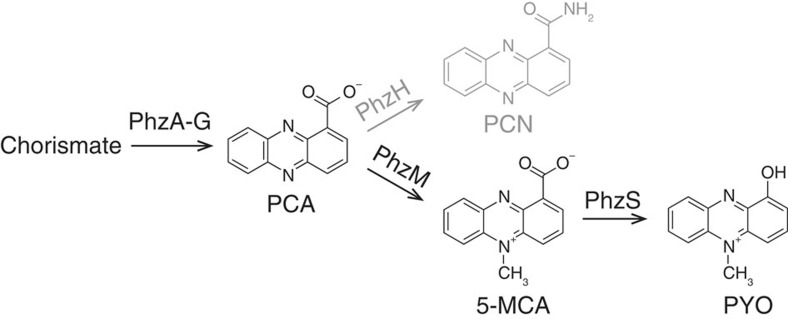
***P. aeruginosa*** **phenazines investigated in this study.** Phenazine biosynthesis, limited to the pathway producing PCA, PCN, 5-MCA and PYO. Arrows are labelled with the phenazine biosynthetic enzymes. PCN is shown in grey as all strains used in this study are Δ*phzH* mutants.

**Figure 2:**
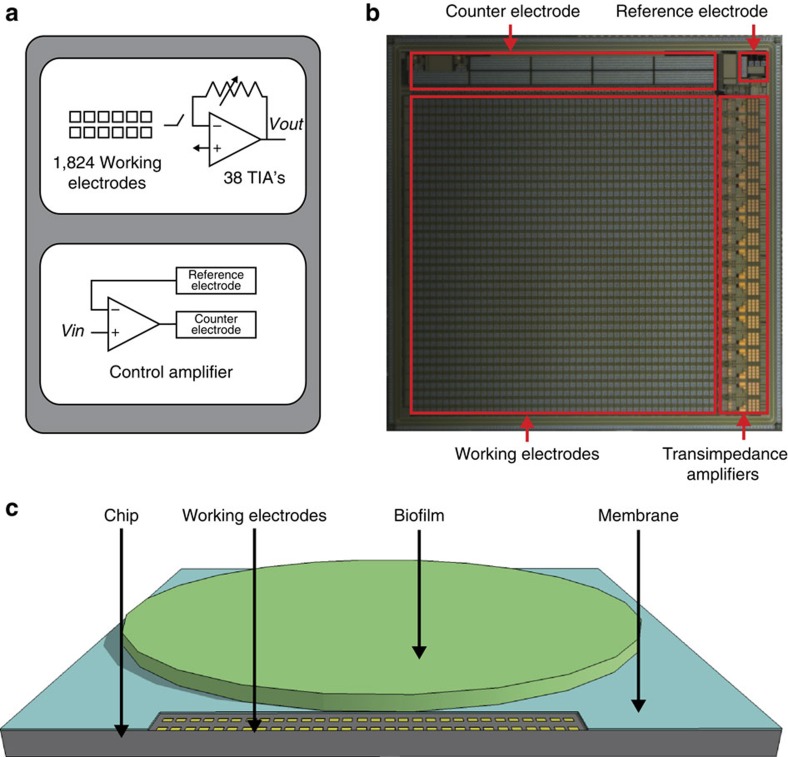
Electrochemical camera imaging platform. (**a**) Block diagram of the electrochemical camera chip. (**b**) Optical micrograph of the electrochemical camera chip with integrated electrodes and amplifiers highlighted. Chip is 1 cm x 1 cm. (**c**) Diagram of the imaging platform with the electrochemical camera chip, working electrodes, biofilm and membrane highlighted.

Next, we ‘add' another phenazine to the studies by imaging a biofilm formed by the Δ*phzHS* strain (Fig. 4a,e) and, thereby, investigate the spatial distribution of PCA conversion to 5-MCA (Fig. 1). We note that 5-MCA is known to be highly reactive^31^ and, therefore, we cannot exclude that a 5-MCA derivative is present^32^. PCA is present at low concentrations throughout the centre of the colony and at slightly higher concentrations at the edge of this area. 5-MCA is detected, with current peaks at −200 mV (reductive SWV) and −150 mV (oxidative SWV) (Fig. 4b,c), at maximum concentration in a ring outside of the PCA-containing area (Fig. 4a,e). This suggests localized activity of PhzM, the methylase responsible for conversion of PCA to 5-MCA. Previous findings suggest that *phzM* expression is cell density-dependent^33^, but analysis of DAPI-stained colony thin sections do not reveal significant variations in cell density relevant for the 5-MCA distribution pattern observed by electrochemical imaging (Supplementary Fig. 14). Given the strict O_2_-dependence of the hydroxylation reaction that converts 5-MCA to PYO, one might expect that O_2_ is also required for PCA methylation and generation of 5-MCA, and that this could explain the localization of 5-MCA to the relatively well-oxygenated region of the colony closer to the edge. However, PCA-addition experiments show that anaerobic cell suspensions catalyse 5-MCA production in a PhzM-dependent manner (Supplementary Fig. 15). We conclude that an alternative unknown parameter affects the conversion of PCA to 5-MCA in the colony centre and gives rise to the ring-shaped distribution of 5-MCA.

**Figure 3:**
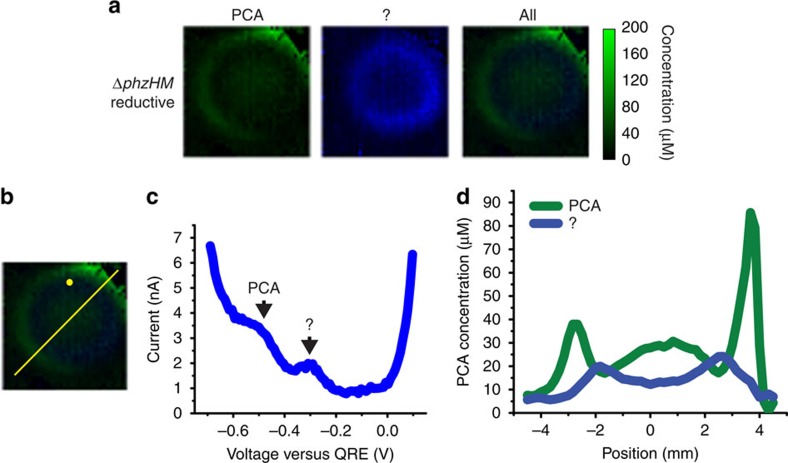
Electrochemical imaging of a Δ*phzHM* biofilm. (**a**) Electrochemical imaging, based on reductive SWV, of a Δ*phzHM* biofilm after 2 days of development. Pixel intensity is proportional to PCA concentration. An unidentified compound provides the reductive peak at −300 mV (**c**). Because the unidentified compound cannot be calibrated to concentration values, it is scaled relative to PCA based on SWV peak current. PCA is shown in green and the unidentified compound in blue. Maximum error due to electrode variability is 28 μM for PCA. Images are 8 mm × 8 mm. (**b**) Locations of example square-wave voltammogram and cross-section in **c**,**d**, respectively. (**c**) Example square-wave voltammogram from a single electrode. (**d**) Example cross-section from the electrochemical image in **a**. Because the unidentified redox-active species cannot be calibrated to concentration values, it is scaled relative to PCA based on SWV peak current.

**Figure 4:**
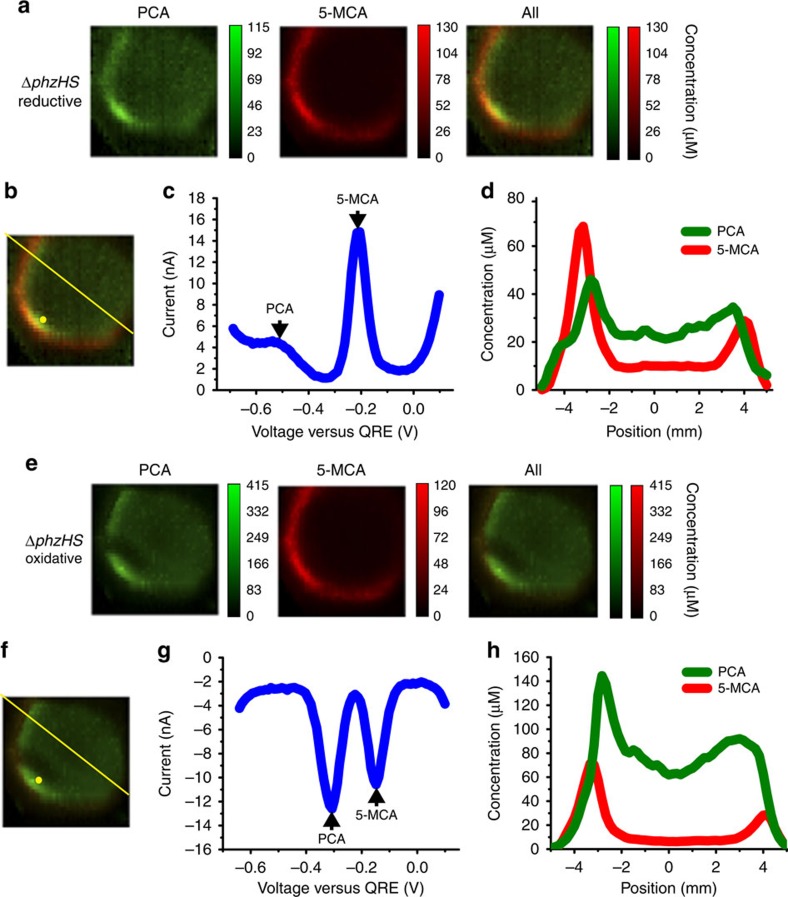
Electrochemical imaging of a Δ*phzHS* biofilm. (**a**) Electrochemical imaging, based on reductive SWV, of a Δ*phzHS* biofilm after 2 days of development Pixel intensity is proportional to phenazine concentration. PCA, green; 5-MCA, red. Maximum error due to electrode variability is 14 μM for PCA and 15 μM for 5-MCA. Images are 8 mm × 8 mm. (**b**) Locations of example square-wave voltammogram and cross-section in **c**,**d**, respectively. (**c**) Example square-wave voltammogram from a single electrode. (**d**) Example cross-section from the electrochemical image in **a**. (**e**) Electrochemical imaging, based on oxidative SWV, of the same Δ*phzHS* biofilm shown in **a**. Pixel intensity is proportional to phenazine concentration. PCA is shown in green and 5-MCA in red. Maximum error due to electrode variability is 74 μM for PCA and 12 μM for 5-MCA. Images are 8 mm × 8 mm. (**f**) Locations of example square-wave voltammogram and cross-section in **g**,**h**, respectively. (**g**) Example square-wave voltammogram from a single electrode. (**h**) Example cross-section from the electrochemical image in **e**.

While the general spatial patterns of oxidized (Fig. 4a,d) and reduced (Fig. 4e,h) PCA and 5-MCA are the same, reduced PCA is present at approximately 3.5 times the concentration of oxidized PCA, while oxidized and reduced 5-MCA are present at nearly identical concentrations. (Supplementary Fig. 16 provides an alternative presentation of the data.) The higher ratio of reduced to oxidized PCA at the edge is suggestive of PCA cellular reduction activity. The different oxidation states of the PCA and 5-MCA pools may arise from differences in reactivity with oxygen or rates of cell-catalysed reduction and await further investigation. Finally, we note that the unidentified phenazine detected in the Δ*phzHM* biofilm (Fig. 3) is no longer detected here. As this phenazine may be an alternate product of PhzA-G activity, we hypothesize that the relative amounts of this compound and PCA are affected by downstream consumption of PCA by PhzM. With 5-MCA synthesis acting as a ‘PCA sink', core phenazine biosynthesis may be shifted from the unidentified compound towards PCA.

**Figure 5:**
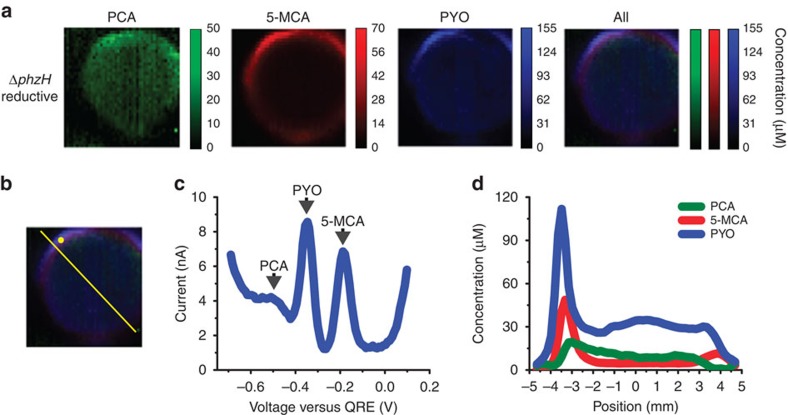
Electrochemical imaging of a Δ*phzH* biofilm. (**a**) Electrochemical imaging, based on reductive SWV, of a Δ*phzH* biofilm after 2 days of development. Pixel intensity is proportional to phenazine concentration. PCA is shown in green, 5-MCA in red and PYO in blue. Maximum error due to electrode variability is 18 μM for PCA, 8 μM for 5-MCA and 14 μM for PYO. Images are 8 mm × 8 mm. (**b**) Locations of example square-wave voltammogram and cross-section in **c**,**d**, respectively. (**c**) Example square-wave voltammogram from a single electrode. (**d**) Example cross-section from the electrochemical image in **a**.

Finally, we image a Δ*phzH* biofilm that produces three phenazines: PCA, 5-MCA and PYO (Fig. 5a). PYO is detected with current peaks at −370 mV (reductive SWV) and −300 mV (oxidative SWV). The overlap of these PCA and PYO peaks means that oxidative SWV can only be used for identification of PCA when PYO is absent. After 2 days of biofilm growth, we find PCA at low levels throughout the centre of the biofilm, while 5-MCA is present at the edge in a ring surrounding the area of PCA production, as is seen in the Δ*phzHS* mutant (Fig. 4). In a similar distribution, PYO accumulates at low levels throughout the centre but is present in higher concentrations at the colony edge, located even outside the ring of high 5-MCA levels. This is consistent with the fact that the conversion of 5-MCA to PYO, catalysed by the monooxygenase PhzS, is an O_2_-dependent reaction^34^, as the outermost edge of the colony is more O_2_-replete than areas within the colony that are further away from the edge^35^.

**Figure 6:**
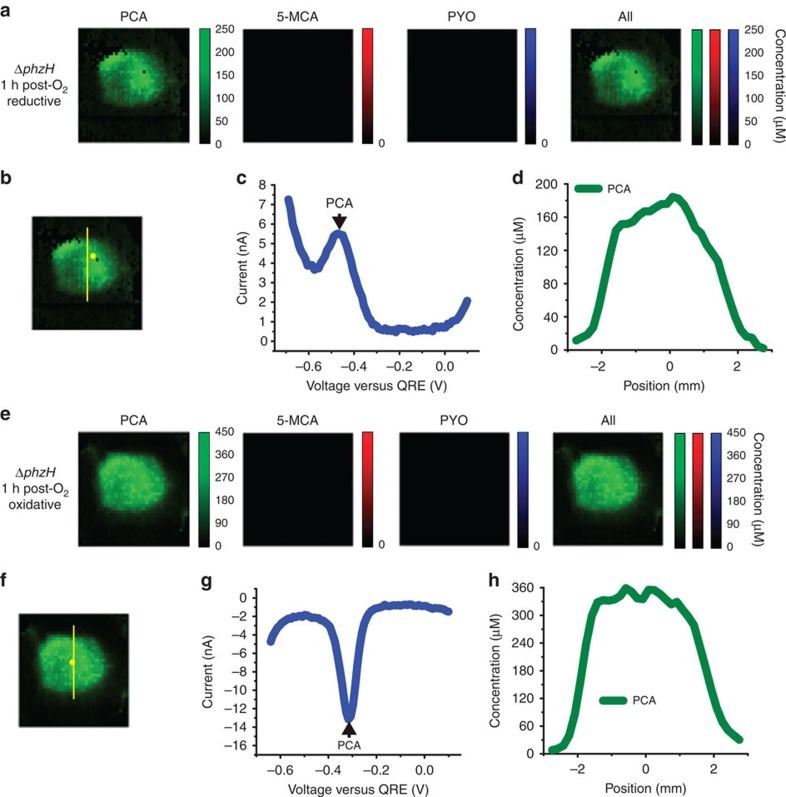
Electrochemical imaging of an anaerobically grown Δ*phzH* biofilm following 1 h of oxygenation. (**a**) Electrochemical imaging, based on reductive SWV, of a Δ*phzH* biofilm, after 2 days of anaerobic development and after 1 h of subsequent exposure to O_2_. Pixel intensity is proportional to phenazine concentration. PCA is shown in green. Maximum error due to electrode variability is 28 μM for PCA. Images are 8 mm × 8 mm. (**b**) Locations of example square-wave voltammogram and cross-section in **c**,**d**, respectively. (**c**) Example square-wave voltammogram from a single electrode. (**d**) Example cross-section from the electrochemical image in **a**. (**e**) Electrochemical imaging, based on oxidative SWV, of the same Δ*phzH* biofilm shown in **a**. Pixel intensity is proportional to phenazine concentration. PCA is shown in green. Maximum error due to electrode variability is 74 μM for PCA. Images are 8 mm × 8 mm. (**f**) Locations of example square-wave voltammogram and cross-section in **g**,**h**, respectively. (**g**) Example square-wave voltammogram from a single electrode. (**h**) Example cross-section from the electrochemical image in **e**.

While Figs 3, 4, 5 demonstrate the utility of the electrochemical camera chip to image spatial distribution of redox-active compounds, we seek to investigate next its ability to study effects of environmental perturbations on these distributions temporally. In addition to the conversion of 5-MCA to PYO, biochemical studies have indicated that the phenazine biosynthetic pathway may contain additional O_2_-requiring steps in the production of PCA (ref. 30). We, therefore, examine the effects of anaerobic growth and subsequent transfer to an aerobic atmosphere on phenazine production and distribution in *P. aeruginosa* colony biofilms. Figure 6 shows SWV-based imaging of a Δ*phzH* biofilm grown anaerobically for 2 days on medium containing nitrate, a respiratory substrate for *P. aeruginosa*, and subsequently exposed to O_2_ for 1 h before imaging. PCA, but neither 5-MCA nor PYO, is detected. As seen in the aerobically grown Δ*phzHM*, Δ*phzHS* and Δ*phzH* biofilms, PCA is present throughout the centre of the biofilm, but the ring of higher concentration, particularly prominent in the aerobically grown Δ*phzHM* colony, is not apparent. The unidentified compound present in the Δ*phzHM*-colony analysis is not detected here, suggesting altered flux towards PCA during growth on nitrate. We speculate that the lack of a higher-concentration PCA ring and the lack of the unidentified compound may both be attributed to the different distribution of electron acceptor in this colony compared with an aerobically grown one. As the electron acceptor for an aerobically grown colony (O_2_) is provided in the atmosphere, it becomes limited at the base and in the colony centre, while nitrate is provided in the growth substrate and is, therefore, available in excess across the entire base of the colony. We note that oxidized PCA is present in higher concentrations than in the aerobically grown Δ*phzHM* colony (Fig. 3). This could be due to non-specific, enzyme-mediated oxidation of PCA by nitrate^36^. (Supplementary Fig. 17 provides an alternative presentation of the data).

**Figure 7:**
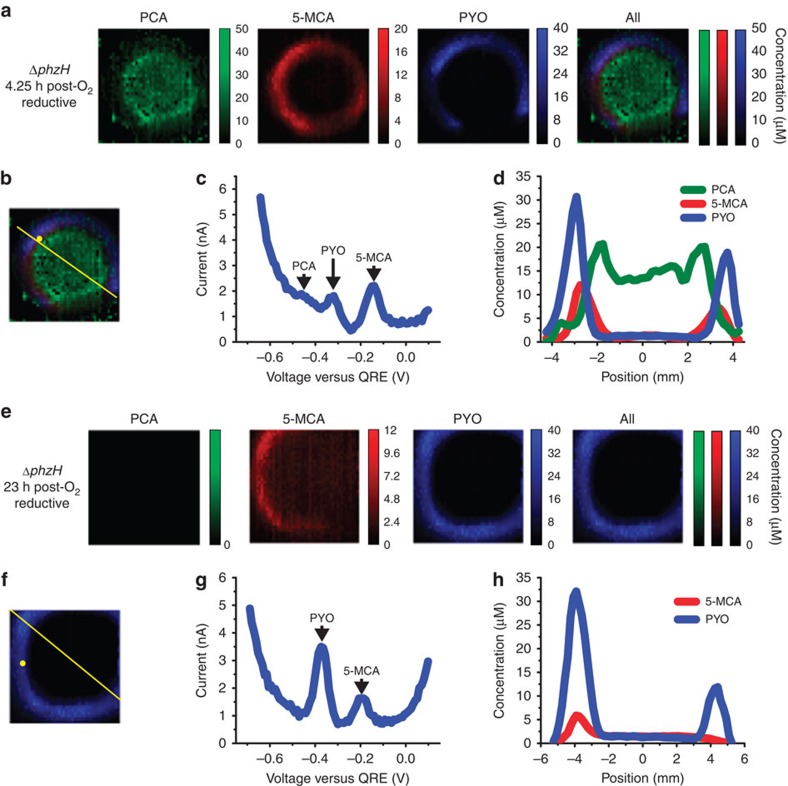
Electrochemical imaging of anaerobically grown Δ*phzH* biofilms following 4.25 and 23 h of oxygenation. (**a**) Electrochemical imaging, based on reductive SWV, of a Δ*phzH* biofilm after 2 days of anaerobic growth and after 4.25 h of subsequent exposure to O_2_. Pixel intensity is proportional to phenazine concentration. PCA is shown in green, 5-MCA in red and PYO in blue. Maximum error due to electrode variability is 18 μM for PCA, 4 μM for 5-MCA and 4 μM for PYO. Images are 8 mm × 8 mm. (**b**) Locations of example square-wave voltammogram and cross-section in **c**,**d**, respectively. (**c**) Example square-wave voltammogram from a single electrode. (**d**) Example cross-section from the electrochemical image in **a**. (**e**) Electrochemical imaging, based on reductive SWV, of a Δ*phzH* biofilm after 2 days of growth and after 23 h of subsequent exposure to O_2_. Pixel intensity is proportional to phenazine concentration. 5-MCA is shown in red, and PYO in blue. Maximum error due to electrode variability is 4 μM for 5-MCA, and 4 μM for PYO. Images are 8 mm × 8 mm. (**f**) Locations of example square-wave voltammogram and cross-section in **g**,**h**, respectively. (**g**) Example square wave voltammogram from a single electrode. (**h**) Example cross-section from the electrochemical image in **e**.

It is somewhat surprising that 5-MCA and PYO are not yet detected at the 1-h time point. After exposing the anaerobically grown Δ*phzH* biofilm to O_2_ for 4.25 h, 5-MCA and PYO are detected at the edge of the colony, with PYO occupying the outermost edge and 5-MCA in a ring located just inside the PYO ring (Fig. 7a–d). The longer aerobic incubation time required for production of these phenazines could be due to slow penetration of oxygen into the colony or a need to activate expression of PhzM and PhzS. At the 4.25-h time point, PCA is still present throughout the centre of the biofilm, but at a lower concentration than after 1 h of aerobic incubation (Fig. 6). After 23 h of O_2_ exposure of an anaerobically grown Δ*phzH* biofilm, PCA levels drop below the detection limit, while PYO levels remain comparable to the 4.25-h time point and 5-MCA is decreased. This suggests a reduced production of total phenazines at the later time point. These results demonstrate the ability of electrochemical imaging to reveal the response of phenazine biosynthetic activity in a colony to changes in the chemical environment.

## Discussion

Multicellularity has evolved in every kingdom of life, and the benefits of multicellularity, such as resistance of biofilms to antimicrobial agents, are well-known^37^. Less well understood are the mechanisms whereby individual cells in a multicellular entity can act together as a community – for example, how a *P. aeruginosa* biofilm coordinates structure formation. This study takes a novel approach towards studying the chemical basis for community behaviour. For any *in vivo* method designed to directly detect metabolites involved in community behaviour, the spatial heterogeneity of physiological conditions within multicellular systems^38^ necessitates a method capable of spatially resolved detection of molecules of interest with high resolution within the intact sample.

While a few IC-based electrochemical arrays have been reported in the literature^39^,^40^,^41^,^42^, to our knowledge, this is the largest electrochemical array with integrated counter and reference electrodes employed in a microbiology context. All of the other arrays employ constant-potential amperometry for any ‘full-frame' measurements. The use of a potential sweep method for full-frame measurements, as applied here, enables simultaneous detection of multiple redox-active species and differentiation between distinct redox states of the same compound, as demonstrated here. The high-resolution afforded by this improved device and technique allows the user to detect features in the metabolite concentration profiles to a resolution better than 250 μm. The high-frame rate supported by our electrochemical camera chip also allows for monitoring the spatiotemporal distribution of redox-active metabolites released from multicellular samples. This makes it amenable to a direct investigation of signalling between cells or even discrete populations of cells, such as spatially separated colony biofilms that are genetically or phylogenetically distinct.

When used to image *P. aeruginosa* PA14 colony biofilms with various capacities for phenazine production and grown under changing conditions, this newly developed electrochemical camera chip provides a view of metabolite distribution that was not previously attainable. These results hint at surprising aspects of phenazine production and metabolism. First, they show that 5-MCA production does not directly correlate with PCA availability (Fig. 4), suggesting that environmental parameters differentially regulate synthesis of these two phenazines. Although we have observed that a ‘coffee ring effect'^12^,^43^ can lead to specific feature formation in PA14 colonies under some conditions, the conditions used to prepare colonies for this study do not lead to obvious differences in cell distribution. This suggests that other environmental parameters affect the conversion of PCA to 5-MCA. Second, although 5-MCA synthesis, which is required for wild-type colony biofilm morphology, is not strictly O_2_-dependent (Supplementary Fig. 15), our analyses raise the possibility that 5-MCA production is nonetheless affected by O_2_ availability as 5-MCA is not detected in anaerobically grown colonies until 4.25 h after they have been exposed to O_2_ (Fig. 7a–h). Furthermore, a greater proportion of the PCA pool is oxidized in the anaerobically grown colony after 1 h of aerobic incubation than in the aerobically grown Δ*phzHS* colony (Supplementary Figs 16 and 17), suggesting that PCA oxidation can be coupled to nitrate reduction under physiological conditions. These findings await further biochemical verification but have implications for our understanding of the roles of phenazine chemistry in biofilm redox homeostasis, morphogenesis and pathogenicity.

As applied here, electrochemical imaging indicates the presence of a phenazine derivative or metabolite that was not detected by other techniques. In its use of SWV, electrochemical imaging is a more ‘open-ended' approach than those that detect current output at a set potential and, therefore, one that may reveal unexpected chemical species in samples. Direct electrochemical probing can be applied for diagnosing and even disrupting biofilm-based infections, as cells assume a biofilm-specific physiological state that contributes to antibiotic resistance. In addition to this potential application in clinical settings, the camera chip developed here could prove useful in characterization of metabolite distribution in naturally formed microbial communities, such as the mats formed by mixed populations of microbes in marshes, as well as disruption of biofilms responsible for decreased efficiency in industrial settings.

The electrochemical imaging approach is also directly applicable to similar studies on eukaryotic systems both *in vivo* and *in vitro*. Studies in eukaryotes have provided countless examples in which compounds (often called morphogens) control the patterning of multicellular structures. More recent work in bacteria has shown that many of the small molecule signals with critical roles in eukaryotic organisms also have regulatory effects in bacterial communities. Nitric oxide, for example, is a potent vasodilator^44^ that has been implicated in dispersal events of biofilms^45^. In addition to distinguishing between metabolites based on their redox potentials, we demonstrate that this method detects different redox states of the same metabolite and can be used to map changes in metabolite distribution after environmental perturbations. Biofilms, such as those found in water distribution systems and the chronic pulmonary infections that occur in patients with cystic fibrosis, persist in industrial and clinical settings with dynamic chemistries. A better understanding of the *in situ* metabolic response to such changes may support future efforts to control their effects. Furthermore, the possibility of locally manipulating biofilms by actively oxidizing or reducing metabolites through the action of specific working electrodes also exists and will be pursued in future work.

## Methods

### Integrated circuit design

The chip is a 1 cm × 1 cm custom IC implemented using a commercial Taiwan Semiconductor Manufacturing Company 0.25 μm CMOS process. The chip was designed and simulated using the Cadence Virtuoso software package.

### Electrode modifications

The working electrodes were initially implemented in aluminium, the top metal layer of the CMOS process. These electrodes were converted to gold using the following process. Photoresist (AZ Electronic Materials, AZ4620) was spun onto the chip using a two-step programme of 300 r.p.m. for 3 s and 4,500 r.p.m. for 45 s, followed by a soft bake at 110 °C for 2 min. The chip was exposed at 12 mW cm^−2^ for 40 s in a mask aligner (SUSS MA6) using a chrome-on-glass mask that defines openings above the working electrodes. For development, the chip was immersed in a 1:3 mixture of developer (AZ Electronic Materials, AZ400K) and water for approximately 120 s. The exposed aluminium was etched in heated aluminium etchant (Transene Aluminum Etchant Type A) for 5 min. Titanium/gold of 4/100 nm thickness was evaporated onto the chip in an electron beam evaporator (Angstrom EvoVac), followed by lift-off in acetone.

Reference electrodes were silver electroplated by immersing electrodes in silver cyanide and applying a sufficient potential between reference electrode sites and a platinum counter electrode to drive 13 mA cm^−2^ through the circuit for 30 min. Following electroplating, silver was converted to silver chloride by immersing the electrodes in bleach diluted 1:10 for 1 min.

### Packaging and supporting electronics

The chip was wire bonded to a 272-pin ball-grid-array package. Dam-and-fill doughnut epoxy encapsulation covered the exposed gold wire bonds, leaving the die surface exposed. The chip was then mounted on a circuit board containing power supply circuitry, a digital-to-analogue converter for inputting the voltage signal during measurements, anti-aliasing filters, multichannel analogue-to-digital converters to convert TIA outputs, and a field-programmable gate array module (Opal Kelly XEM6010) with a Universal Serial Bus interface for the transfer of digital signals to and from the chip and printed circuit board.

### CMOS chip electrochemical measurements

On-chip electrochemical cells consisted of on-chip gold working electrodes, an integrated gold counter electrode and an integrated silver/silver chloride electrode serving as a quasireference electrode in a three-electrode potentiostat configuration. The feedback loop established the excitation voltage signal on the reference electrode. The working electrodes were maintained at a constant potential and were connected to TIA's to read the output current. The SWV excitation signal consisted of a staircase ramp with 60 mV forward steps and 50 mV reverse steps at 33 Hz frequency. The voltage range of the input signal was 0.1 to −0.7 V. The TIA was set to a gain of 10 MΩ.

After each colony was grown to the desired time point according to the procedures described below, it was transferred to the chip. For each colony measurement, a piece of track-etched membrane (Whatman 110606, pore size=0.2 μm) with a biofilm growing on top was placed on the chip following wetting of the membrane bottom with liquid agar. After 1.5 min, square-wave voltammograms were measured at all 1,824 electrodes, with each of the 48 columns of electrodes connected to the 38 output channels one after another. Total measurement time for all 1,824 electrodes was 5.2 min. Colonies remained on the chip for the 1.5 min waiting period and 5.2 min measurement period. During this time period, small agar droplets were applied to the agar layer when necessary to counteract drying. The output current signal from a SWV experiment consisted of the response to a series of forward and reverse voltage steps. To create the square-wave voltammogram, the 10 current samples at the end of each reverse voltage step were averaged and subtracted from the 10 averaged current samples at the end of each forward voltage step. The resulting signal was plotted as a function of the forward applied potentiostat voltage. For quantification of peak heights, the peak current was measured from a fitted baseline. Current values are converted to concentration values using the calibration experiments in Supplementary Figs 4–6. The following procedure was used to compute maximum error due to electrode variability. In calibration experiments, electrodes in the array were exposed to known concentrations of phenazines and the current at each electrode was measured. The measured currents were averaged and plotted versus concentration to produce calibration curves. The s.d. from the mean of current was also calculated for each concentration value. The maximum s.d. among all the s.d.'s was chosen and converted to concentration using the calibration curve to produce the maximum error due to electrode variability value. In electrochemical images, values at malfunctioning electrodes (that is, electrodes lacking a reasonable electrochemical output signal, average 5%) were interpolated from surrounding electrode values. Cross-sections were computed in ImageJ using the Plot Profile feature and a line width of 10.

### Preparation of colonies

Liquid cultures were grown in lysogeny broth^46^ at 37 °C with shaking at 250 r.p.m. overnight. The overnight cultures were diluted 1:50 in lysogeny broth and grown to exponential-early stationary phase. Colonies used for on-chip electrochemical measurements were prepared by spotting 5 μl of exponential-early stationary phase culture onto a track-etched membrane (Whatman 110606, pore size is 0.2 μm) placed on a 1% agar, 1% tryptone plate containing 40 μg ml^−1^ Congo red and 20 μg ml^−1^ Coomassie blue. The colonies were grown at 25 °C, >95% humidity in an incubator (Percival) for the desired time. Colonies grown anaerobically were spotted on track-etched membranes placed on 1% agar, 1% tryptone plates containing Congo red, Coomassie blue and 40 mM potassium nitrate. The colonies were grown at room temperature in a vinyl anaerobic chamber with an atmosphere of 80%/15%/5% N_2_/CO_2_/H_2_ (Coy).

## Additional information

**How to cite this article:** Bellin, D. L. *et al*. Electrochemical camera chip for simultaneous imaging of multiple metabolites in biofilms. *Nat. Commun.* 7:10535 doi: 10.1038/ncomms10535 (2016).

## Supplementary Material

Supplementary Information Supplementary Figures 1-17
