# Supplementary material for: Electrochemical camera chip for simultaneous imaging of multiple metabolites in biofilms
Source: Nat Commun. 2016 Jan 27;7:10535. doi: 10.1038/ncomms10535 (PMC4737866; doi:10.1038/ncomms10535)
Supplement: Supplementary Information — Supplementary Figures 1-17 [file ncomms10535-s1.pdf]

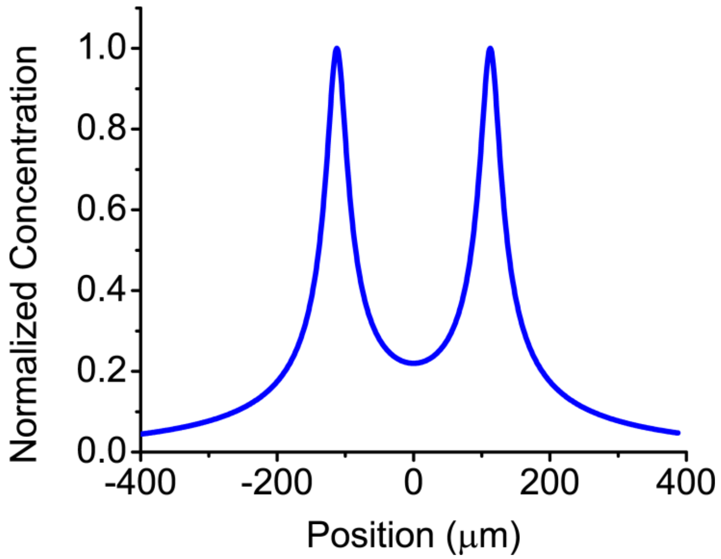

Supplementary Figure 1.

Diffusion profile for PYO from two semi-spherical sources of radius 1 µm spaced 225 µm apart and located 15 µm above, assuming a diffusion coefficient of 0.5 x 10^{-9} m^2 s^{-1}.

We have carefully assessed the effect of diffusion on achievable spatial resolutions. Biofilms are grown on track-etched membranes to facilitate movement of biofilms from thick agar plates onto the chip. Prior to placement on the chip, the bottom of the membrane is wet with liquid agar. Based on optical profilometry measurements, we estimate the agar to be, at most, 15 µm thick.

In general, analysis of the effects of diffusion on the performance of the imager follows from the solution of the three-dimensional diffusion equation for the concentration *C(x,y,z,t)*: $\partial C/\partial t = D\nabla^2 t$ in a rectangular agar slab with the top surface defined by z=0 and the bottom surface by *z*=*W*, where *D* is the diffusion coefficient. An initial condition *(t*=0*)* with *C*=0 everywhere is assumed. Zero-flux boundary conditions are assumed at the top and bottom.

For *t*>0, if the concentration in a semi-sphere surface of radius a centered at $x=x_o$, $y=y_o$, $z=0$ is assumed to have a fixed concentration of C_s, the concentration at the bottom of the slab due to the action of this source at time t is given by $P(x, y, t) = 2 \cdot C_s \cdot \frac{a}{r} \cdot efrc\left(\frac{r-a}{2\sqrt{Dt}}\right)$ where $r = \sqrt{(x - x_0)^2 + (y - y_0)^2 + W^2}$. The method of images is used to model the zero-flux boundary conditions for the finite-thickness agar slab into which the molecules spread.

We have previously reported a diffusion coefficient of 0.5 x 10^{-9} m^2 s^{-1} for PYO[11]. For an approximate experiment time of 6.5 min and an agar thickness of 15 µm, the diffusion profile from two semi-spherical sources 225 µm apart (the pitch of the on-chip electrodes) is shown here. As is evident, because the agar thickness is considerably thinner than the diffusion length, each source is well-resolved, indicating that electrode geometry, and not diffusion, limits resolution in this measurement platform.

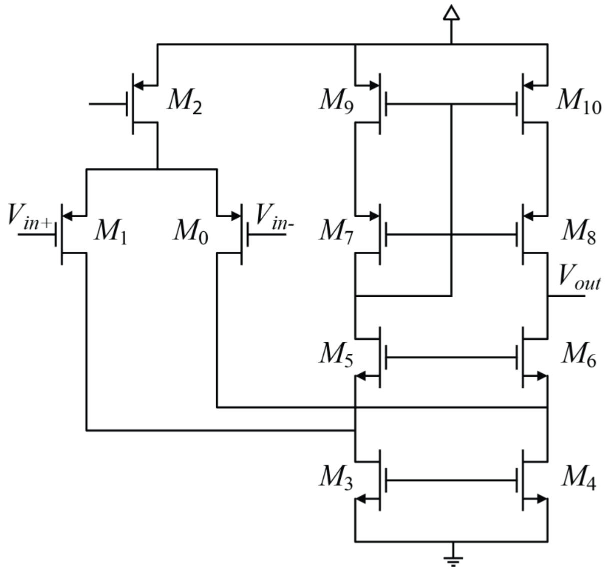

Transistor	Width / Length (µm)
M_0 / M_1	1200 / 0.9
M_2	300 / 0.6
M_3 / M_4	210 / 0.7
M_5 / M_6	85 / 1.0
M_7 / M_8	140 / 0.6
M_9 / M_{10}	280 / 0.6

Supplementary Figure 2.

Circuit diagram of the OTA

The integrated circuit (IC) consists of the following components: a working electrode array, a control amplifier establishing a three-electrode potentiostat configuration, a reference electrode, a counter electrode, and parallel output channels of transimpedance amplifiers (TIA's).

The IC features a 48 x 38 array of 1824 working electrodes, each 100 x 100 µm^2 in size, originally implemented as aluminum in the top metal layer of the semiconductor process. The aluminum electrodes are later replaced with gold as described in the Online Methods. A column decoder takes a 6-bit input address word and selects one of the 48 columns of electrodes, connecting each of the 38 electrodes in that column to an individual TIA. All other electrodes not selected by the decoder are connected to external bias pins on the circuit board.

The IC features 38 parallel TIA’s outputting data from the chip. The input to each TIA is one working electrode from the working electrode array, and the transimpedance amplifier converts the current flowing through the working electrode to a voltage for output from the chip. Each transimpedance amplifier is implemented as a folded-cascode operational transconductance amplifier (OTA) and feedback impedance. The OTA has a simulated gain of 73 dB around a 1.65-V bias point. The transconductance amplifier circuit of this circuit is shown above with transistor sizes shown in the inset table.

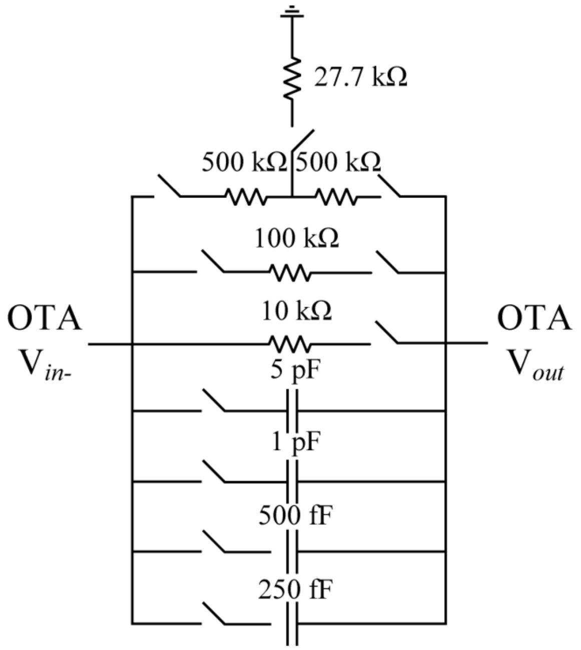

Supplementary Figure 3.

Transimpedance amplifier feedback impedance circuit.

The programmable feedback impedance of the transimpedance amplifier contains selectable resistors in parallel with capacitors. The available resistances, equivalent to the available current-to-voltage gains, are 10 kΩ, 100 kΩ, 1 MΩ, or 10 MΩ. To select the 1 MΩ resistance, switches close to place two 500 kΩ resistors in series. To select the 10 MΩ resistance, switches close to implement a T-network of two 500-kΩ resistors in series, between which is a 27.7-kΩ resistor terminating to ground. The available capacitors in the feedback impedance are 5 pF, 1 pF, 500 fF, or 250 fF.

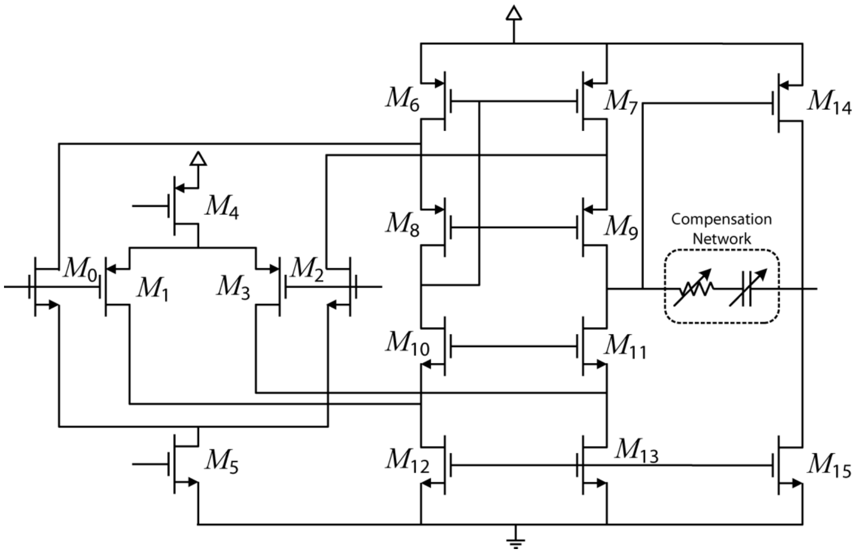

Transistor	Width / Length (µm)
M_0 / M_2	280 / 0.7
M_1 / M_3	500 / 0.6
M_4	1140 / 0.7
M_5	210 / 0.7
M_6 / M_7	780 / 0.6
M_8 / M_9	260 / 0.6
M_{10} / M_{11}	60 / 0.7
M_{12} / M_{13}	170 / 0.7
M_{14}	1450 / 0.6
M_{15}	300 / 0.7

Supplementary Figure 4.

Control amplifier circuit.

The IC features a control amplifier to establish a three-electrode potentiostat configuration. The control amplifier takes an input voltage signal at its positive terminal, a reference electrode at its negative terminal, and a counter electrode at its output terminal. The control amplifier is implemented as a two-stage operational amplifier consisting of a dual-input, folded-cascode first stage and a common-source second stage. The control amplifier has a gain of approximately 103 dB and a second-stage bias current of 3 mA. The control amplifier circuit is shown in the figure with the transistor sizes given in the inset table.

There is a programmable RC-compensation network between the two stages of the control amplifier. The compensation network contains selectable 100-Ω, 1-kΩ, or and 10-kΩ resistors in series with a 24-pF capacitor.

The reference and counter electrodes can be chosen to be on-chip electrodes or external electrodes connected to pins on the circuit board. The on-chip reference electrode is 0.08 mm^2 in area and the on-chip counter electrode is 5 mm^2 in area, both implemented as aluminum in the top metal layer of the semiconductor process. As discussed in Online Methods, both on-chip electrodes are replaced with gold, and the reference electrode is further converted to a silver/silver-chloride quasireference electrode.

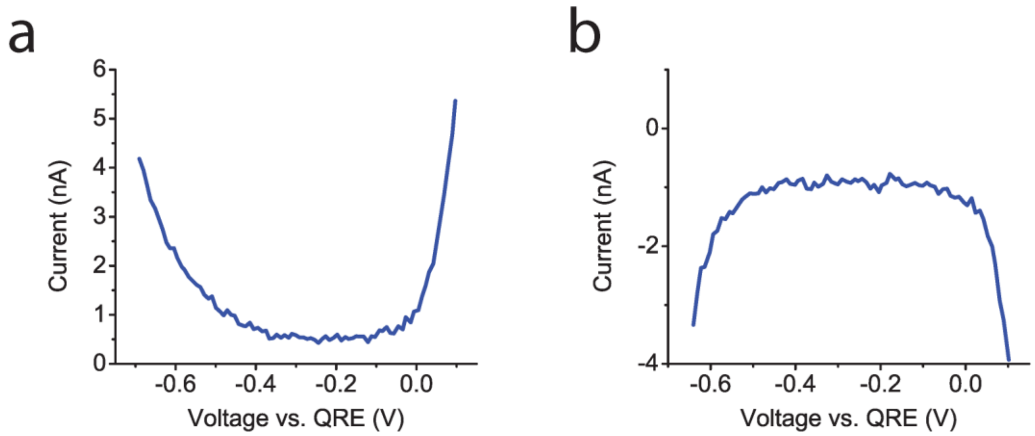

Supplementary Figure 5.

Square wave voltammagrams for electrodes across a Δ*phz* biofilm after two days of growth: (a) reductive and (b) oxidative.

This measurement establishes a "baseline" for biofilms producing no phenazines.

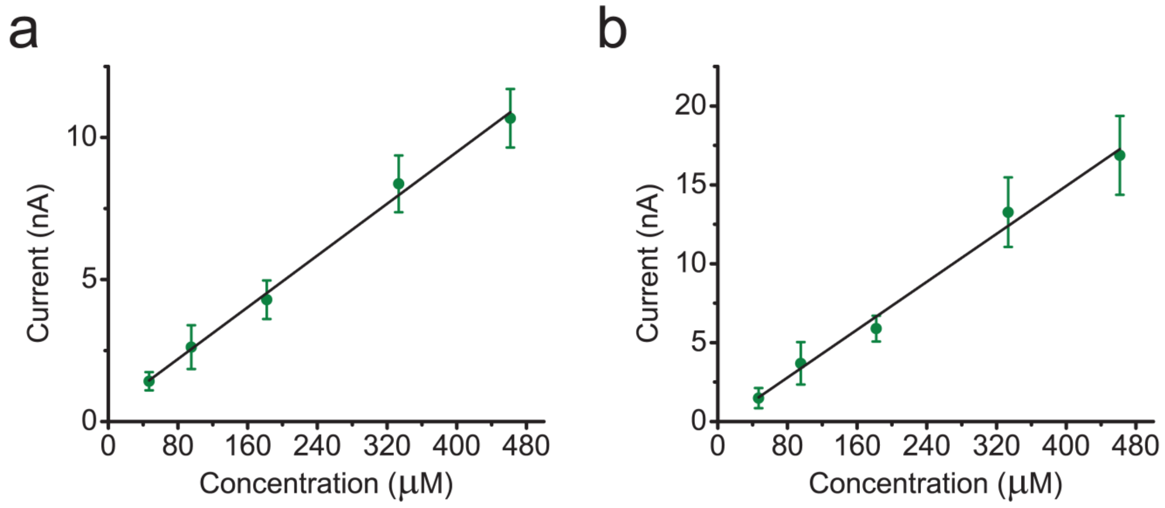

Supplementary Figure 6.

Calibration curves for synthetic PCA on (a) reductive and (b) oxidative SWV.

Current measurements are calibrated to concentration values using linear fitting to results from dilution series of synthetic PCA. Current measurements were averaged over 120 electrodes in the chip array.

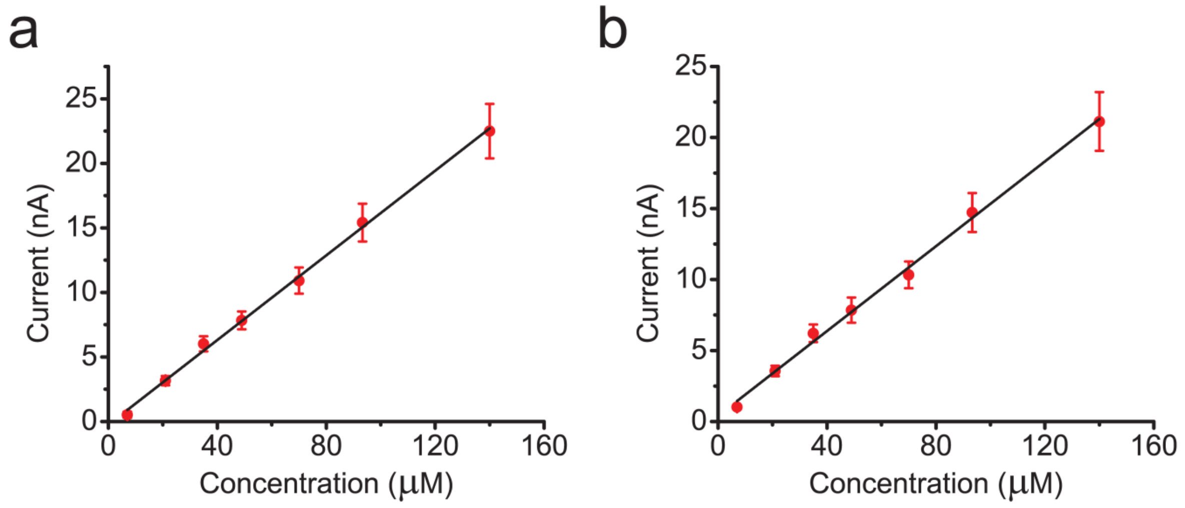

Supplementary Figure 7.

Calibration curves for purified 5-MCA on (a) reductive and (b) oxidative SWV.

Current measurements are calibrated to concentration values using linear fitting to results from dilution series of purified 5-MCA. Current measurements were averaged over 120 electrodes in the chip array.

To obtain purified 5-MCA, Δ*phzHS* strain is inoculated from a streaked plate in 5 mL MOPS medium containing 50 mM MOPS buffer, 43 mM NaCl, 93 mM NH_4Cl, 2.2 mM KH_2PO_4, 1 mM $MgSO_4$•7H_2O, 3.6 μM $FeSO_4$•7H_2O, 20 mM glucose, and 20% LB. The culture is grown at 37 ˚C shaking at 250 r.p.m. for 17 hours. The culture is centrifuged and the supernatant passed through a 0.2 μm filter. 50 microlitres of supernatant is loaded onto a Waters Symmetry C-18 reverse phase column (Waters; 5 μm particle size, 4.6 x 150 mm^2). The phenazines are separated using a gradient of water-0.01% TFA (solvent A) to acetonitrile-0.01% TFA (solvent B) at a flow rate of 0.4 ml min^{-1} using the following protocol: linear gradient from 0 to 15% solvent B for 2 min, linear gradient to 83% solvent B for 20 min, linear gradient from 83 to 100% solvent B for 10 min and finally, a linear gradient to 0% solvent B for 5 min. The total method time is 38 min. The retention time for 5-MCA is 6.2 min. The detection wavelength is 366 nm. The concentration of 5-MCA is determined by taking the area under the peak at the 6.2 min elution time using System Gold 32 Karat Software (Beckman Coulter) and a conversion factor for PYO (8 x 10^{-6} μM AU^{-1}).

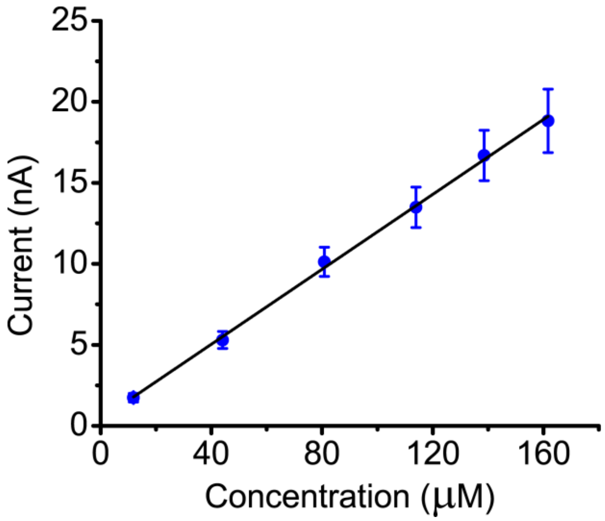

Supplementary Figure 8.

Calibration curve for synthetic PYO on reductive SWV.

Current measurements are calibrated to concentration values using linear fitting to results from dilution series of synthetic PYO. Current measurements were averaged over 120 electrodes in the chip array.

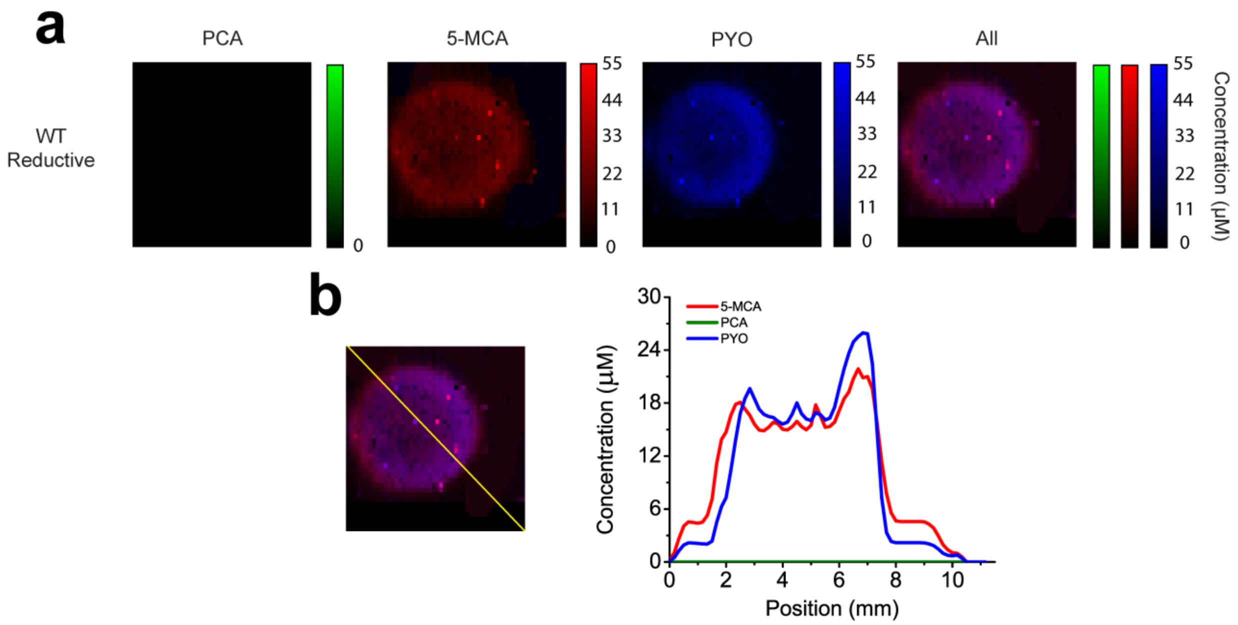

Supplementary Figure 9.

Electrochemical imaging of a wild-type biofilm.

The above (a) images and (b) cross-section were measured for a 30-hour old wild-type colony under the same conditions used to image the Δ*phzH* colony shown in Fig. 5. The distribution of PYO and 5-MCA show similar peaking at the colony edges. PCA is not detected in significant amounts, due to its overlap with the broad PCN peak and/or increased flux to downstream phenazine derivatives relative to the Δ*phzH* mutant.

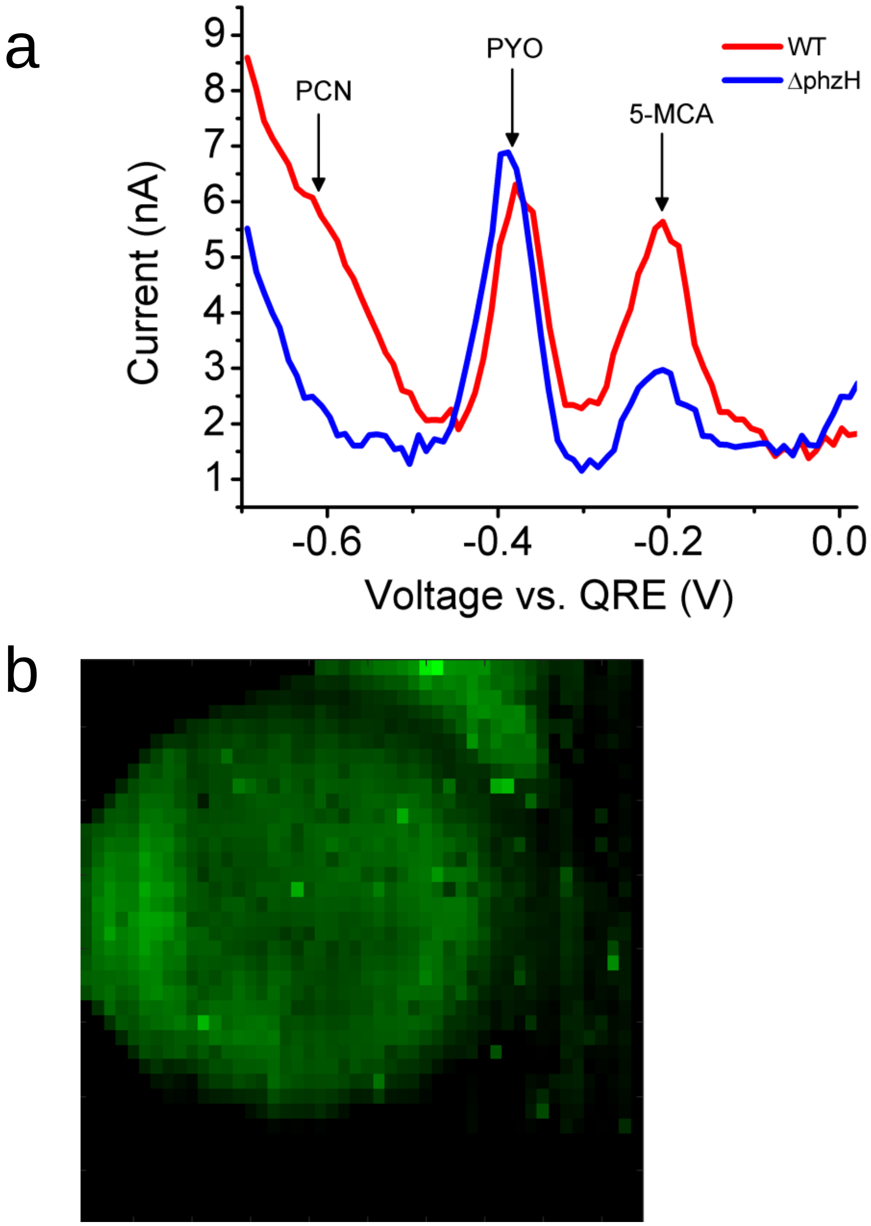

Supplementary Figure 10.

On-chip square wave voltammogram from wild-type (WT) and Δ*phzH* biofilms, and an image of SWV current due to PCN in a WT colony.

The above figure demonstrates the ability of the chip to detect PCN, which is produced by *P. aeruginosa* PA14 WT but not by Δ*phzH*. (a) PCN produces a peak in positive-to-negative SWV at approximately -600 mV vs. QRE. (b) An image of PCN peak SWV current in a 30-hour old WT colony. Maximum peak current in this image is 8 nA. PCN appears to be produced uniformly through the colony.

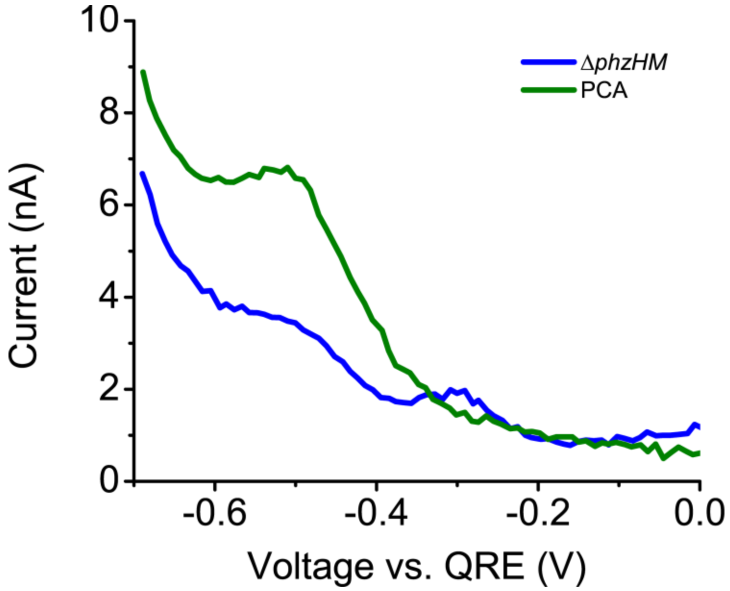

Supplementary Figure 11.

Square wave voltammograms from the Δ*phzHM* biofilm of Figure 3 and from synthetic PCA.

An overlay of reductive SWV traces indicates that the peak at -500 mV, rather than the peak at -300 mV, is due to PCA. The latter peak is absent from reductive SVW traces generated for the Δ*phz* biofilm (Supplementary Fig. 5) and may represent another product of the PhzA-G-dependent portion of the phenazine biosynthetic pathway.

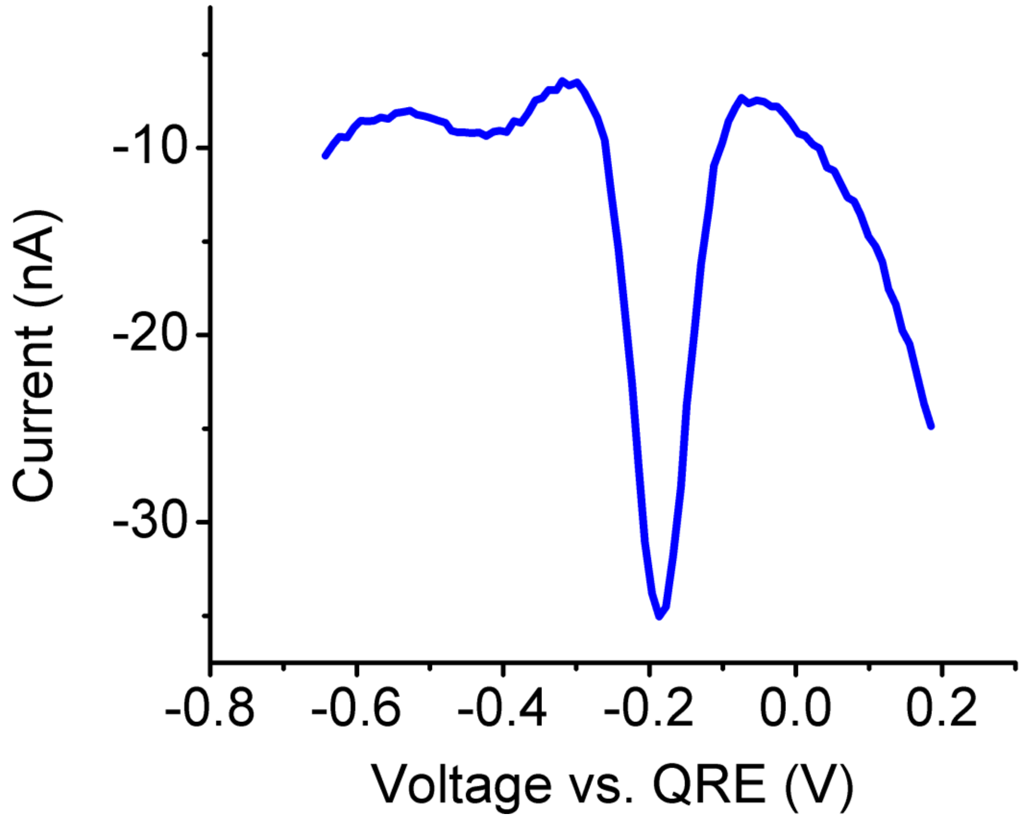

Supplementary Figure 12. Square wave voltammogram of phenazine-1,6-dicarboxylic acid (PDC).

The above on-chip square wave voltammogram was measured for phenazine-1,6-dicarboxylic acid (PDC) (Tractus Chemical, 85% purity) dissolved in 1% tryptone to a final concentration of 3 mM. The peak due to PDC is at -200 mV vs. QRE, which is different from the -300 mV vs. QRE peak due to the unidentified phenazine in Fig. 3. This indicates that the unidentified phenazine is not PDC.

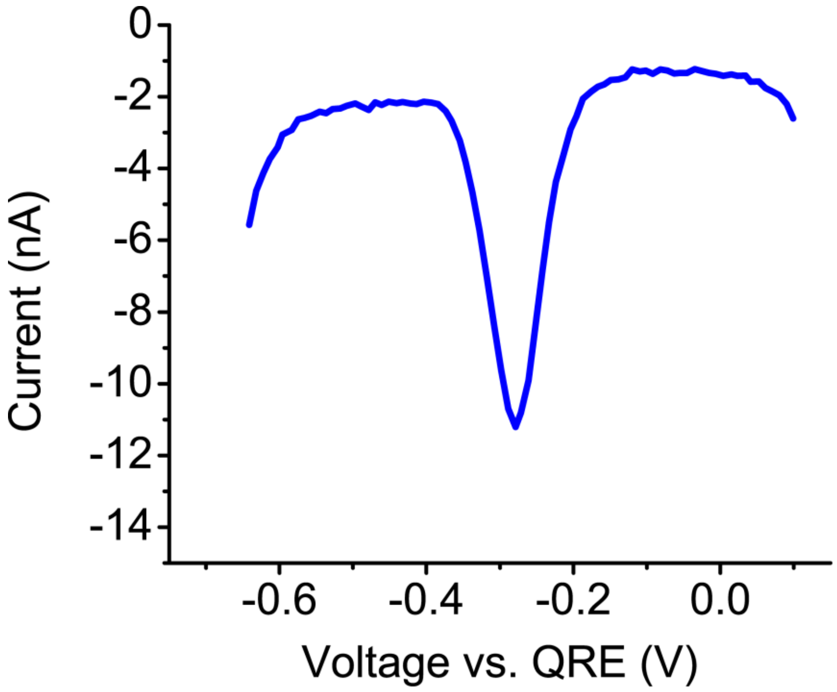

Supplementary Figure 13.

Oxidative square wave voltammogram from the Δ*phzHM* colony of Fig. 3.

The peak generated by the unidentified compound, seen at -300 mV on reductive SWV, overlaps with the PCA peak in oxidative SWV traces at -300 mV.

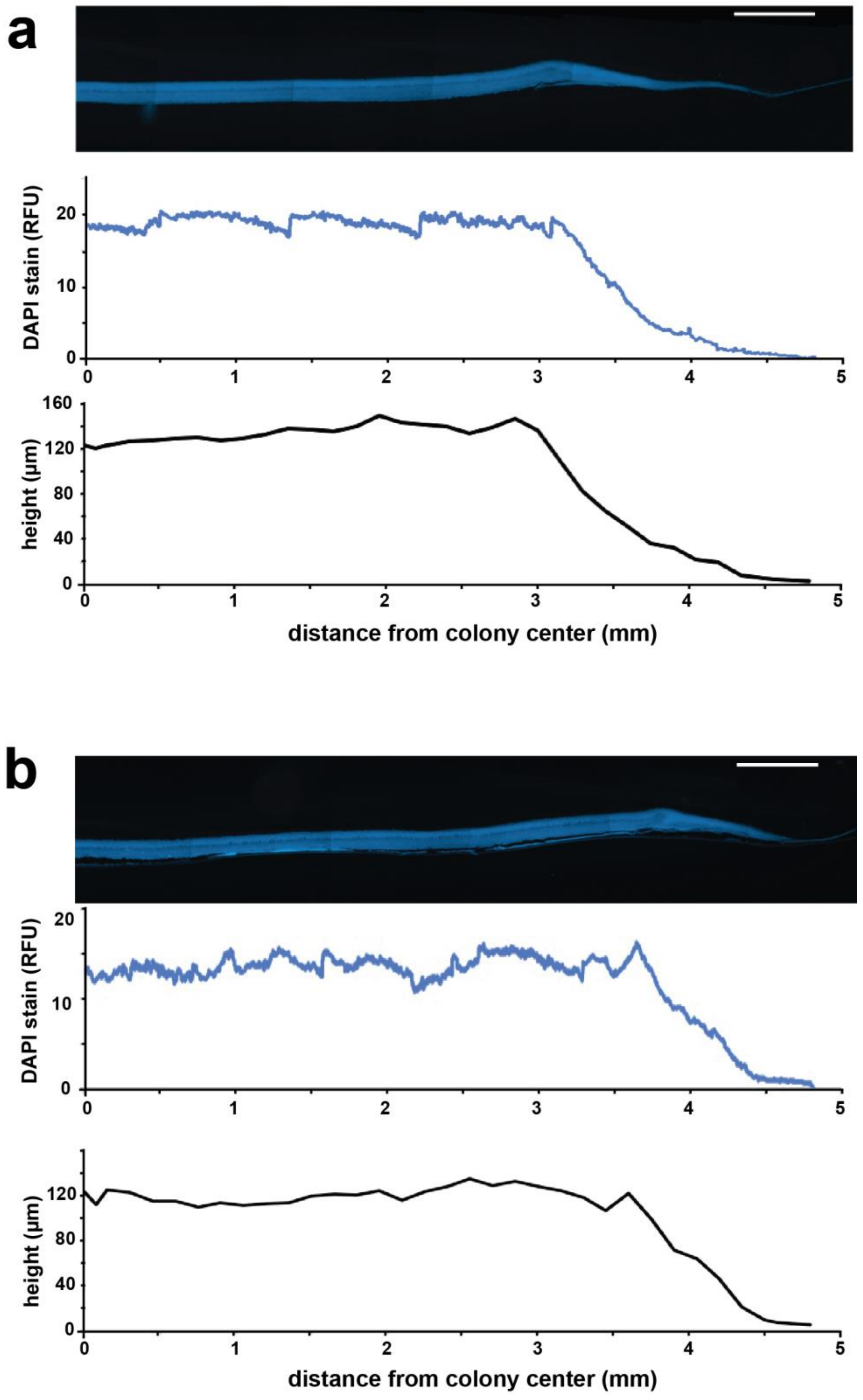

Supplementary Figure 14.

Colony thin sections of (a) wild-type and (b) the Δ*phzH* mutant strain.

Sections were stained with DAPI and imaged using Zeiss AxioZoomV.16 macroscope (excitation = 365 $_{BP}$/ 50nm, emission = 445 $_{BP}$/ 50nm). Image depicts one representative of three different sections ranging from colony center to edge. DAPI fluorescence and height measurements were plotted. Scale bars are 500 µm.

Preparation of colony thin sections for fluorescence microscopy

Plates for colony growth were prepared by pouring 1% tryptone, 1% agar containing 20 µg/mL Coommassie blue and 40 µg/mL Congo red to a depth of 4 mm and allowing media to solidify. Cultures were inoculated into 2mL lysogeny broth (LB) and shaken overnight at 250rpm and 37°C in the dark. Overnight cultures were diluted 1:50 in fresh LB and sub-cultured for ~160 min before adjusting to an OD (500nm) of 0.5-0.8. Immediately prior to spotting, 25-mm polycarbonate membrane filters (Whatman, VWR) were laid across the plates with the matte sides facing up. Relevant strains were then spotted onto the center of the filters in 5µL inoculants and cultured in a humidified chamber at 25°C. One colony was spotted per filter. After growth, filters were lifted from the media and excess filter was trimmed from around the colonies. Filters were then laid across a 1.5-mm layer of polymerized 1% agar in a petri dish. An additional 1.5-mm layer of agar was poured over the colonies and allowed to polymerize, laminating the colonies and the filter between two layers of agar. These were cut and lifted from the plate, placed into histosettes (Fisher Scientific) and fixed for 24 hours in a mixture of 2% paraformaldehyde and 2.5% glutaraldehyde containing 0.15% L-lysine and 0.1% DMSO (pH 7.4). Fixed colonies were then subjected to two 90-minute washes in phosphate buffered saline (PBS) before dehydration through a series of ethanol washes [25%, 50%, 70%, 95%, and 100% (three times per concentration)]. Colonies were then cleared via three 90-minute incubations in Histoclear-II (National Diagnostics, Fisher Scientific). Cleared colonies were infiltrated via two 3-hour incubations in 100% paraffin wax (Paraplast Xtra, Fisher Scientific) at 55˚C. All dehydration, clearing, and infiltration steps were carried out using a STP120 Tissue Processor (Thermo Fisher Scientific). Infiltrated colonies were then cast into polystyrene molds and allowed to polymerize overnight at 4˚C. Samples were removed from the molds and paraffin blocks were trimmed using a razor blade. Trimmed blocks were sectioned (10 µm-thick sections perpendicular to the plane of the biofilm) on a Thermo Microm355S rotary microtome using low-profile blades (Sturkey, Fisher Scientific). Sections were floated onto a water bath at 48˚C and collected on frosted slides (Fisher Scientific). Slides were air-dried overnight, heat-fixed on a hotplate for 1 hour, and rehydrated in the reverse order of processing described above, to PBS. Rehydrated colonies were immediately mounted in a 1:10 DAPI:Fluorogel solution (Electron Microscopy Sciences, Fisher Scientific), cover-slipped, and sealed using clear nail polish.

Fluorescence detection of 4',6-diamidino-2-phenylindole (DAPI) was performed using an AxioZoomV.16 macroscope (Zeiss, Germany) under the following filter configurations: excitation = 365 $_{BP}$/ 50nm, emission = 445 $_{BP}$/ 50nm. Digital images were captured with an AxioCam MRm camera attached to the AxioZoomV.16 using the Zen Blue Software (Zeiss).

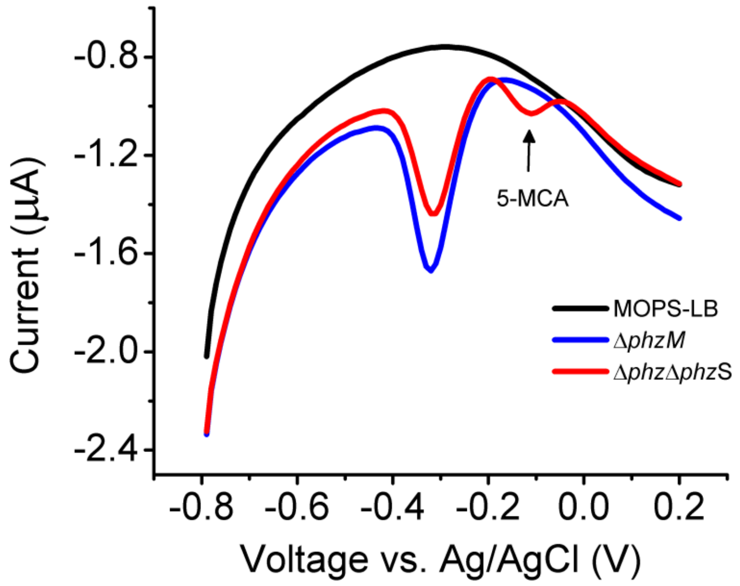

Supplementary Figure 15. Anaerobic cell suspensions catalyze 5-MCA production in a PhzM-dependent manner

Overnight cultures of Δ*phz*Δ*phzS* and Δ*phzM* PA14 were diluted inside an anaerobic chamber into stoppered and crimped Balch tubes containing 5 mL MOPS-LB medium (50 mM MOPS buffer, 43 mM NaCl, 93 mM NH_4Cl, 2.2 mM KH_2PO_4, 1 mM $MgSO_4$•$7H_2O$, 3.6 µM $FeSO_4$•$7H_2O$, 20 mM glucose, 20% LB) supplemented with 100 µM PCA. The cultures were grown for 16 hours at 37 ˚C shaking at 250 rpm in the dark. The supernatant was collected from 1 mL of the culture by centrifuging at 16873 x g for 2 minutes. Phenazines in the supernatant were analyzed using a commercial potentiostat (CH Instruments, CHI760D) with an electrochemical cell consisting of a 1.6 mm-diameter gold working electrode, a platinum counter electrode, and a Ag/AgCl reference electrode (BASI). The SWV input signal consisted of a staircase ramp from -0.8 to 0.2 V, with 10 mV increments, 50 mV stair amplitude and 10 Hz frequency.

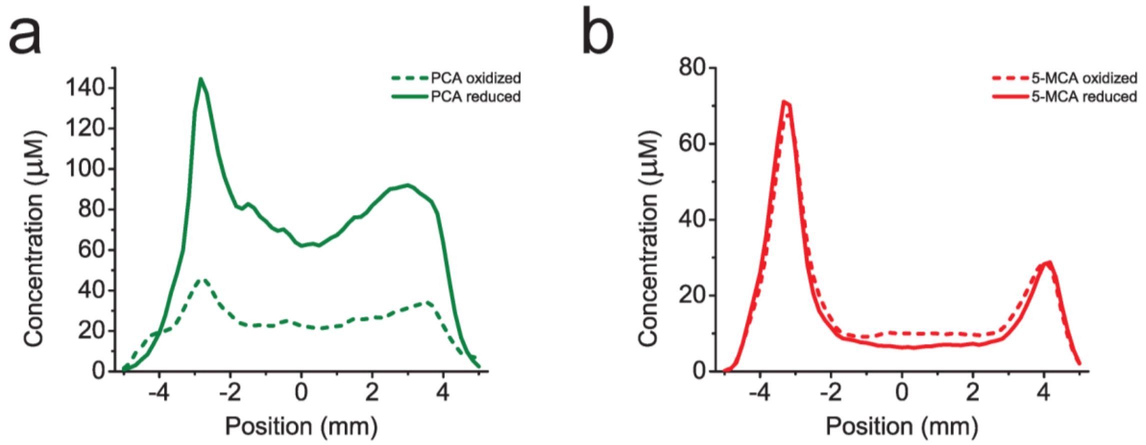

Supplementary Figure 16.

Cross-sections for the Δ*phzHS* colony of Fig. 4, with the reductive and oxidative results for (a) PCA and (b) 5-MCA overlaid.

While the general spatial patterns of oxidized and reduced PCA and 5-MCA are the same, reduced PCA is present at approximately 3.5 times the concentration of oxidized PCA, while oxidized and reduced 5-MCA are present at nearly identical concentrations.

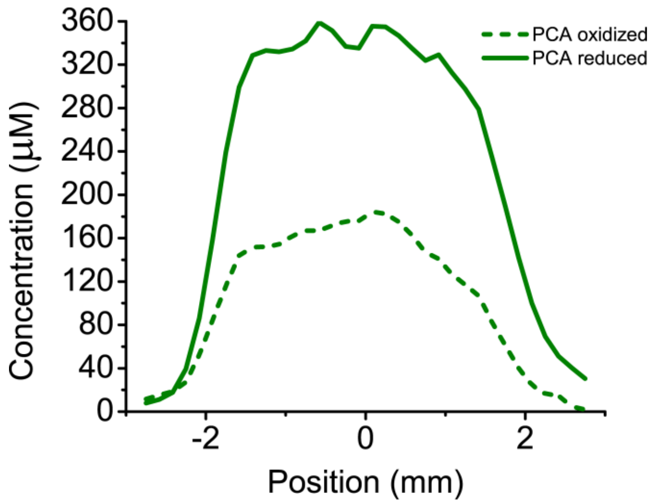

Supplementary Figure 17.

Cross-sections for the anaerobically-grown Δ*phzH* biofilm after 1 hour of oxygenation of Fig. 6, with the reductive and oxidative results for PCA overlaid.

While the general spatial pattern of oxidized and reduced PCA is the same, reduced PCA is present at approximately two times the concentration of oxidized PCA.
